# Vitamin B_12_ Metabolism: A Network of Multi-Protein Mediated Processes

**DOI:** 10.3390/ijms25158021

**Published:** 2024-07-23

**Authors:** Patryk Mucha, Filip Kus, Dominik Cysewski, Ryszard T. Smolenski, Marta Tomczyk

**Affiliations:** 1 Department of Biochemistry, Medical University of Gdansk, 80-210 Gdansk, Poland; patryk.mucha@gumed.edu.pl (P.M.); kusfi@gumed.edu.pl (F.K.); rt.smolenski@gumed.edu.pl (R.T.S.); 2 Laboratory of Protein Biochemistry, Intercollegiate Faculty of Biotechnology of University of Gdansk and Medical University of Gdansk, 80-307 Gdansk, Poland; 3 Clinical Research Centre, Medical University of Bialystok, 15-276 Bialystok, Poland; dominik.cysewski@umb.edu.pl

**Keywords:** vitamin B_12_, metabolism, proteins, anemia, neurodegeneration

## Abstract

The water-soluble vitamin, vitamin B_12_, also known as cobalamin, plays a crucial role in cellular metabolism, particularly in DNA synthesis, methylation, and mitochondrial functionality. Its deficiency can lead to hematological and neurological disorders; however, the manifestation of these clinical outcomes is relatively late. It leads to difficulties in the early diagnosis of vitamin B_12_ deficiency. A prolonged lack of vitamin B_12_ may have severe consequences including increased morbidity to neurological and cardiovascular diseases. Beyond inadequate dietary intake, vitamin B_12_ deficiency might be caused by insufficient bioavailability, blood transport disruptions, or impaired cellular uptake and metabolism. Despite nearly 70 years of knowledge since the isolation and characterization of this vitamin, there are still gaps in understanding its metabolic pathways. Thus, this review aims to compile current knowledge about the crucial proteins necessary to efficiently accumulate and process vitamin B_12_ in humans, presenting these systems as a multi-protein network. The epidemiological consequences, diagnosis, and treatment of vitamin B_12_ deficiency are also highlighted. We also discuss clinical warnings of vitamin B_12_ deficiency based on the ongoing test of specific moonlighting proteins engaged in vitamin B_12_ metabolic pathways.

## 1. Introduction

**Figure 1:**
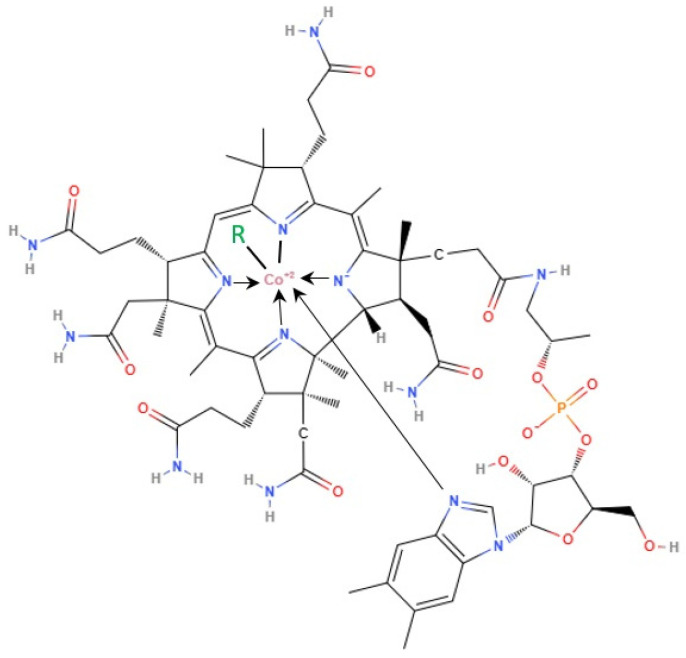
Skeletal formula of cobalamin. R (green) = CN, HO, methyl, or 5-deoxyadenosyl, N (blue)—nitrogen, Co^+2^ (pink)—cobaltous cation, O (red)—oxygen, H (grey)—hydrogen, P (orange)—phosphorus, arrows—possible bindings.

Vitamin B_12_ (cobalamin) was initially isolated in 1948 by two independent scientific teams—Folker’s in the USA and Smith’s in Great Britain—and identified: it was previously described as the antipernicious anemia factor [1]. Structurally, it possesses an organometallic nature, with the cyanide ion coordinated to the central cobalt atom. The primary Co ligand is surrounded by a corrin macrocycle containing four reduced pyrrole rings and seven amide side chains [2]. The precise structure varies among different corrinoids, where vitamin B_12_ is the only form active in animals and humans. The pyrrols constitute the equatorial plane of the ring, which is supplemented by two axial Co-ligands: a nucleotide base firmly attached to the ring and an exchangeable external ligand, e.g., water, cyanide, and methyl- or 5′-deoxyadenosyl group (as well as a number of other nucleophiles), see Figure 1. Hydroxycobalamin and cyanocobalamin are metabolically inactive; however, upon conversion to methylcobalamin and 5-deoxyadenosylcobalamin, the vitamins attain full activity [3]. Among these exchangeable groups, water represents the weakest coordination ligand, while CN-group exhibits the strongest coordination [4].

**Figure 2:**
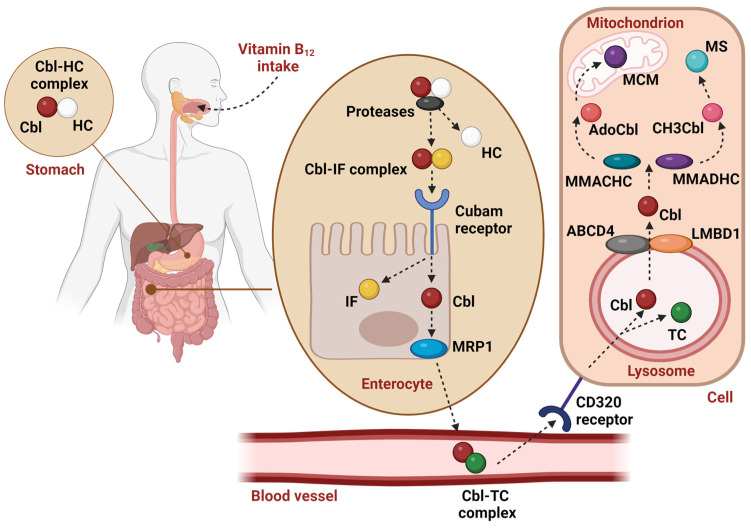
Adsorption, blood transport, and intracellular metabolism of vitamin B_12_. Cbl—cobalamin, HC—haptocorrin, IF—Intrinsic Factor, TC—transcobalamin II, MRP1—multidrug resistant protein 1, ABCD4—ATP binding cassette subfamily D member protein, LMBD1—lysosomal cobalamin transport escort protein, MMACHC—methylmalonic aciduria and homocystinuria type C protein, MMADHC—methylmalonic aciduria and homocystinuria type D protein, AdoCbl—adenosylcobalamin, CH3Cbl—methylcobalamin, MCM—methylmalonyl CoA mutase, MS—methionine synthase. Created with BioRender.com.

Humans cannot endogenously synthesize cobalamin and, consequently, must obtain it through dietary intake, predominantly sourced from meat, fish, dairy products, eggs, or some edible algae symbiotic with vitamin B_12_-producing microorganisms. Diminished intake or impaired absorption of vitamin B_12_ is usually caused by a decreased synthesis of a specific intestinal vitamin B_12_-carrier Intrinsic Factor (IF), see Figure 2, and the disease manifests itself in elderly individuals or in vegans adhering to dairy and egg-free diets [5]. In herbivores, the intestinal uptake of vitamin B_12_ is supported by the gut microbiota; whereas, in all carnivores and omnivores (including humans) the internal production of vitamin B_12_ is of very limited importance compared to dietary sources. Not all corrinoids produced in nature find their biological function in human metabolism and some of them might even disrupt the normal metabolism of this vitamin [6]. One of the examples is Coα-[α-(7-adenyl)]-Coβ-cyanocobamide, a corrinoid isolated from *Lactobacillus reuteri* and commonly named pseudovitamin B_12_. This analog shares a structure resembling cobalamin, except for the lower cobalt ligand. In this modification, nucleotide base adenine substitutes the 5,6-dimethylbenzimidazole [7]. Even though pseudovitamin B_12_ does not inhibit the uptake of vitamin B_12_ in rats [8], some studies suggest that it could potentially disrupt the absorption of a physiological trace concentration of vitamin B_12_ in the medium through the occupation of the vitamin B_12_-transporting blood protein transcobalamin (TC, the product of gene *TCN2*) [9].

The overall transportation chain of vitamin B_12_ is roughly outlined in Figure 2. The absorption of dietary vitamin B_12_ is preceded by the release of cobalamin bound to food proteins. First, gastric acid and proteases take action in the stomach. Afterward, a salivary protein called haptocorrin (HC, the product of gene *TCN1*) binds to liberated vitamin B_12_. The protein moiety of the HC-vitamin B_12_ complex is subsequently degraded by the proteases of the upper small intestine. Next, in the proximal ileum, cobalamin creates a complex with Cobalamin Binding Intrinsic Factor (IF), which is further recognized by certain receptors (first of all, the receptor cubam). Finally, the absorbed vitamin B_12_ is released from IF and excreted into the blood, where it is captured by TC [10]. The “active” transporter TC has a fast turnover rate and, under a steady state, caries about 20% of the vitamin B_12_ in the circulation [5]. The subsequent cellular uptake of vitamin B_12_ involves the CD320 receptor, present in all cell types [11]. Next, the vitamin B_12_-TC complex is transferred into lysosomes where the protein carrier breaks down. ATP Binding Cassette Subfamily D Member 4 protein (ABCD4, encoded by the gene of the same name) and lysosomal cobalamin transport-escort protein (LMBD1, *LMBRD1* gene) are responsible for its further transport from the lysosomal lumen to the cytosol. When cobalamin is released into the cytosol, the cytoplasmic chaperons methylmalonic aciduria and homocystinuria type C protein (CblC, gene *MMACHC*) and methylmalonic aciduria and homocystinuria type D protein (CblD, gene *MMADHC*) initiate processing of the incoming vitamin, which starts with its reduction to Co^2+^ accompanied by removal of the original exchangeable group [12]. In the cytoplasm, vitamin B_12_ is converted to methylcobalamin (CH3Cbl, MeCbl), while adenosylcobalamin (AdoCbl) is synthesized in the mitochondria. MeCbl acts as a carrier of methyl groups. It is a co-factor of the enzyme methionine synthase (MS, gene *MTR*), which is involved in the transformation of homocysteine to methionine in the cytosol. These processes and subsequent metabolic reactions are involved in the synthesis of neurotransmitters, phospholipids, DNA, and RNA. On the other hand, AdoCbl acts as a cofactor of a mitochondrial enzyme, methylmalonyl-coenzyme A mutase (MCM, gene *MMUT*), which converts methylmalonyl-CoA to succinyl-CoA. This reaction is involved in the catabolism of cholesterol, fatty acids, and several amino acids [11]. The impairment of those enzymatic reactions leads to the elevation of homocysteine and methylmalonic acid, non-metabolizable end products, which is important in vitamin B_12_ deficiency diagnosis.

Due to its involvement in various cellular pathways (including isolation of nerve axons and DNA synthesis), alterations in vitamin B_12_ metabolism strongly correlate with cognitive impairment, hematological disorders, and cardiovascular diseases [13,14]. Thus, more in-depth research is necessary to fully elucidate the steps and compounds involved in vitamin B_12_ metabolic pathways.

This review aims to comprehensively summarize all of the identified proteins essential for the proper metabolism of vitamin B_12_ in humans and diseases related to its dysfunction. Furthermore, based on the collected data, we propose new diagnostic options as well as new warnings within the field of vitamin B_12_ deficiency research.

## 2. Proteins Involved in Vitamin B_12_ Metabolism

### 2.1. Gastrointestinal Vitamin B_12_ Transport

#### 2.1.1. Haptocorrin (HC) and Intrinsic Factor (IF)

As previously elucidated, the absorption and utilization of vitamin B_12_ within the body require its initial binding to carrier proteins in the gastrointestinal tract. In humans, the two proteins facilitating this binding process are haptocorrin (HC) and Intrinsic Factor (IF). Despite their immunological distinctiveness, they exhibit structural similarities due to their primary and secondary protein structures [15,16]. In humans, parietal cells within the gastric mucosa of the ileum produce Intrinsic Factor (IF). The gene responsible for encoding IF is known as Gastric Intrinsic Factor (*GIF*) or Cobalamin Binding Intrinsic Factor (*CBLIF*), which is situated on chromosome 11 (11q12.1) in humans and on chromosome 19 in mice [17,18]. IF is made of 399 amino acid residues [16] and has a two-domain organization, with each domain having a mass of approx. 30 kDa and 20 kDa (including the conjugated sugar residues). Vitamin B_12_ is bound to the two domains via multiple bonds [16].

Initially named the R-binder or transcobalamin I (TCN1), haptocorrin (HC) is the first carrier protein for ingested vitamin B_12_. The investigation of the *TCN1* gene sequence and its localization suggests that HC is an evolutionary product of *CBLIF* duplication [19]. This glycoprotein of 60 to 70 kDa [16,19] is present in various body fluids including saliva, breast milk, bile, tears, and blood plasma [17,20]. Upon ingestion, vitamin B_12_ is bound by HC secreted in saliva, which is responsible for transferring the free vitamin to the stomach. HC also binds dietary vitamin B_12_, released from food proteins in the stomach [17]. In the acidic pH of the stomach, HC is thought to protect the vitamin from partial hydrolysis [21]. Vitamin B_12_ remains bound to HC at the entrance to the duodenum, where pancreatic proteases, namely trypsin, chymotrypsin, and elastase, degrade HC, allowing for the release of vitamin B_12_ and its subsequent binding by IF, which was produced in the stomach but remained incapable of binding vitamin B_12_ until the acidic medium became neutralized [11]. Interestingly, deficiencies of pancreatic proteases can lead to defects in vitamin B_12_ absorption due to the inability to degrade HC and release the vitamin from the vitamin B_12_–HC complex. Patients with exocrine pancreatic insufficiency or chronic pancreatitis, conditions marked by inadequate delivery of pancreatic digestive enzymes to the intestine, often develop vitamin B_12_ malabsorption. However, these conditions rarely lead to severe vitamin B_12_ deficiency [22,23,24].

Studies on the exact role of HC in humans are limited due to the lack of homologous genes encoding HC in both mice and rats [25]. In these rodents, salivary glands produce another vitamin B_12_-binding protein transcobalamin (TC), which in humans is responsible for binding the vitamin in blood plasma [11]. This points out that details of the binding of ingested vitamin B_12_ vary between different species, where only some of them express HC [25].

After the release of vitamin B_12_ from HC in the duodenum, vitamin B_12_ binds to Intrinsic Factor (IF). IF is mostly found in gastric juice and ileal fluid in humans, being produced and secreted by parietal cells in the stomach. It is responsible for transferring vitamin B_12_ along the small intestine to the intestinal epithelial cells, which are mainly composed of enterocytes [16,21]. When vitamin B_12_ is bound to IF, its domains can be assembled. This mechanism ensures that only the IF–vitamin B_12_ complex, not the apoprotein present in a high excess, can be recognized and absorbed with the help of the specific receptor cubam [15,26,27].

IF deficiency can be caused by the presence of anti-IF or anti-parietal cell autoantibodies as well as the age-dependent or autoimmune degeneration of parietal cells, e.g., autoimmune gastritis (AIG). The presence of autoantibodies is often linked to *Helicobacter pylori* (*H. pylori*) infection. Patients with *H. pylori* infection have been found to have decreased vitamin B_12_ levels in serum [28]. The exact cause of AIG is still unknown and there is no treatment available. The wide range of unspecific clinical symptoms, which are often subtle (many patients are asymptomatic for a long period after the disease onset), result in difficulties in diagnosing the condition promptly [29]. The lack of IF in this condition leads to long-lasting vitamin B_12_ deficiency due to the inability to deliver vitamin B_12_ to the cells within the ileum and subsequent failure to distribute it throughout the body. The consequence of vitamin B_12_ deficiency in AIG is pernicious anemia (PA) [11,30]. Around 10–15% of patients with AIG develop PA, characterized by hematological, neurological impairments, or both. The hematological manifestation includes megaloblastic anemia, while the clinical neurologic manifestations of PA include weakness, depression, impaired nerve function, cognitive impairment, and sensory deficits [11,31].

#### 2.1.2. Cubam Receptor

When the vitamin B_12_–IF complex reaches the intestine, it is internalized into enterocytes by receptor-mediated endocytosis. Cubam is a multiligand apical membrane receptor expressed in absorptive epithelia in several tissues. In the kidney, cubam is expressed in the proximal tubular epithelium where it mediates the reabsorption of proteins from the renal filtrate, such as albumin, apolipoprotein A-1, hemoglobin, transferrin, and vitamin-D binding protein, leading to reduced proteinuria [32,33]. Cubam is also present in the visceral yolk sac, where it is essential to coordinate maternal nutrient uptake and proper embryo development [34,35]. In the epithelium of the distal ileum, the only known function of cubam is to facilitate the uptake of the IF–vitamin B_12_ complex [33].

Cubam is composed of two proteins: cubilin (CUBN) and type-1 transmembrane protein amnionless (coded by gene *AMN*). Cubilin is a 460 kDa protein that contains three distinct regions: an N-terminal domain, responsible for membrane association; an extracellular fragment formed by epidermal growth factor-like repeats; and 27 domains of complement components C1r/C1s, epidermal growth factor-related protein 1 (Uegf), and bone morphogenetic protein 1 (Bmp1), CUB [36]. Four CUB domains, CUB_5–8_, are responsible for the interaction with the IF–cobalamin complex, as revealed by crystal structures [37]. The remaining domains are involved in the recognition and binding of other ligands, for example, during ultrafiltration and reabsorption in the kidney [38]. To form a receptor, three CUBN-subunits combine in a pin-shaped molecule that docks to transmembrane protein AMN. Cubilin lacks a transmembrane domain and, therefore, the interaction with amnionless is necessary for the formation of a functional cubam complex [32].

AMN-protein is a 45-kDa transmembrane protein also composed of three parts: an extracellular domain, a transmembrane helix, and a cytoplasmic domain responsible for clathrin-mediated internalization. In addition to anchoring CUBN-protein to the apical membrane of epithelia, amnionless is also required for biosynthetic processing and trafficking of cubilin to the plasma membrane. It also mediates cubam receptor recycling and endocytic signaling [39]. The cytoplasmic domain of amnionless features two putative FXNPXF endocytic signals that closely resemble the FXNPXY signal found in the low-density lipoprotein receptor (LDLR) superfamily. The related signaling involves clathrin-associated sorting of proteins (CLASPs) and promotes the clathrin-dependent internalization of various cubam ligands in an analogous way to the internalization of LDLRs [33]. The LDLR family also includes the protein Megalin, which strongly colocalizes with cubam, especially in the kidney where they cooperate in ligand recognition. However, in the intestine, cubam appears to be functioning independently of Megalin, suggesting that only AMN and CUBN protein moieties are required to form a receptor specific for IF–vitamin B_12,_ internalization [40].

Mutations in either of the *CUBN* or *AMN* genes disrupt cubam receptor functioning and result in malabsorption of vitamin B_12_ and the development of Imerslund–Gräsbeck syndrome (IGS). IGS is a rare autosomal recessive disorder that appears in childhood. It is characterized by megaloblastic anemia as a consequence of vitamin B_12_ deficiency [41]. Most IGS patients also develop mild proteinuria and non-specific symptoms attributed to vitamin B_12_ deficiency [42], even though, if left untreated, the disease may result in neurological impairments and can be fatal. Treatment with parenteral vitamin B_12_ therapy, typically via regular intramuscular injections, is effective in managing the condition [43,44]. Recent data also demonstrate altered expression of amnionless in the kidney and the ileum with aging. This causes vitamin B_12_ deficiency irrespectively of its normal nutritional uptake as well as the development of age-related chronic diseases [45].

#### 2.1.3. Multidrug Resistant Protein 1 (MRP1)

Upon the uptake to the enterocyte, the next step in vitamin B_12_ transport is its distribution to other cells and tissues in the body. To achieve this, vitamin B_12_ needs to be exported into the blood circulation and this step is mediated by multidrug resistant protein 1 (MRP1, gene *ABCC1*).

MRP1 has been described for the first time, in 1992 by Cole et al. This 190 kDa glycoprotein that has been shown to export a wide range of substrates including bioactive compounds, signaling molecules, metabolites, anti-cancer drugs, and xenobiotics, thus conferring multidrug resistance in cancer. For example, MRP1 is overexpressed in many hematological and solid tumors [46,47]. Under physiologic conditions, MRP1 is ubiquitously expressed in the body, mostly in the lung, testis, kidney, heart, placenta, small intestine, colon, brain, and peripheral blood mononuclear cells [47,48,49]. In the small intestine, MRP1 is mainly present in the basolateral membranes of the crypt cells [50]. In addition to the plasma membrane localization, MRP1 has also been shown to accumulate in subcellular organelles such as endocytic vesicles, trans-Golgi vesicles, and lysosomes [48,51], although its involvement in the intracellular transport of vitamin B_12_ has never been demonstrated. MRP1, similarly to lysosomal vitamin B_12_ exporter ABCD4, also belongs to the ABC transporters family, hence its alternative name ABCC1 [46].

MRP1 has been shown to efflux vitamin B_12_ from the intestinal epithelium to the blood circulation [52]. Mice deficient in MRP1 (*MRP1^(−/−)^*) have decreased levels of vitamin B_12_ in their plasma, simultaneously accumulating the vitamin in the ileum and colon. These studies also established that MRP1 transports the vitamin in its protein-free form, contrary to the previously hypothesized export of the vitamin in complex with transcobalamin [52,53]. In some patients with recessive hereditary vitamin B_12_ malabsorption, mutations in the MRP1-encoding gene *ABCC1* were detected; however, they were ruled out as potential causes of vitamin B_12_ deficiency as they either appeared in the intronic gene fragments, which did not affect the open reading frame, or did not fulfill Mendelian rules for inheritance [54]. These studies were conducted on a small group (approx. 30) of patients, suggesting a need for large-scale epidemiological investigation to clarify the involvement of *ABCC1* mutations in the development of vitamin B_12_ deficiency. Such investigations could potentially validate the redundancy in vitamin B_12_ export given the existence of alternative pathways aside from MRP1 export in humans. Among other closely related transporters of the ABC family with wide tissue distribution (ABCB6, ABCG2, MRP3, and MRP5), only MRP1 was shown to be involved in vitamin B_12_ efflux [52]. Moreover, as presented by Beedholm-Ebsen et al., *MRP1^(−/−)^* mice have not presented morphologic and metabolic abnormalities, suggesting that other transporters should compensate for the lack of MRP1 (also considering a passive transport of vitamin B_12_ through membranes herein).

### 2.2. Proteins Involved in Vitamin B_12_ Blood Transport

#### Transcobalamin (TC)

When vitamin B_12_ is exported into the blood circulation, it is bound by a 43 kDa protein transcobalamin, a former transcobalamin II (gene *TCN2*) [55]. In humans, TC is synthesized by a variety of cell types and tissues and secreted to blood plasma [56,57]. TC is structurally related to IF and HC, with some conserved regions sharing 60–80% homology; all three vitamin B_12_ transporters are derived from a common ancestral gene [53,58]. TC is responsible for the distribution of vitamin B_12_ into all of the cells in the body but only about 25% of the total vitamin B_12_ in plasma is bound by TC, while the rest is carried by circulating HC [59,60]. The complex of vitamin B_12_ and transcobalamin is referred to as holoTC and is the “active” form of vitamin B_12_ with a fast turnover rate, making vitamin B_12_ available to cells. In contrast, the biological function of the complex with HC (holoHC) is not clear [21].

HoloTC levels in serum have been suggested as a specific marker of early vitamin B_12_ deficiency diagnosis with the use of commercially available immunoassays for holoTC [61]. However, some studies indicate that this is a controversial determinant of vitamin B_12_ status. Some patients with severe vitamin B_12_ deficiency, resulting in PA, displayed falsely normal holoTC levels [62]. It was also hinted that the rationale behind the diagnostic holoTC immunoassay is based on a false assumption that holoTC is the first factor to respond to vitamin B_12_ deficiency, where the level of methylmalonic acid (MMA), a form of substrate for methylmalonyl-CoA mutase that requires vitamin B_12_ as a cofactor, and is more sensitive to vitamin B_12_ deficits in the early stage [63,64].

Mutations in the *TCN2* gene can hamper or even prevent intracellular accumulation of cobalamin, causing a rare multisystemic disorder characterized by autosomal recessive inheritance with symptoms such as pancytopenia, megaloblastic anemia, developmental delay, diarrhea, psychomotor impairment, and immune system deficiency [65]. Only a few cases have been investigated so far, showing either a large deletion of the entire exon 8 leading to a premature stop codon (p.E371fsX372) [66] or a change in intron 4 leading to the loss of the start codon as a cause of gene alternation [67].

### 2.3. Proteins Involved in Intracellular Vitamin B_12_ Transport

#### 2.3.1. CD320 Receptor

CD320, also known as the receptor for cobalamin-saturated transcobalamin (TCblR), is a small 282 amino acid protein with 29 kDa calculated molecular weight (observed 58 kDa, probably due to a high glycosylation) [68]. It belongs to the LDLR protein family. CD320 is localized on the cell surface and contains two domains connected by the transmembrane helix. These are the C-terminal cytoplasmic region and the extracellular N-terminal fragment with an epidermal growth factor (EGF) separating the two LDLR type A sites [69,70]. A soluble form (sCD320) was also found in the human serum fraction [71]. CD320 exhibits high specificity and affinity toward transcobalamin and the TC–cobalamin complex is bound to CD320 and absorbed by the cells in the presence of Ca^2+^ ions [72,73]. Initially, holo-TC binds to the N-terminal region of CD320 whereupon it is internalized into an endocytic vesicle. Next, endosomes with holo-320 complexes are fused with a lysosome, where TC and CD320 undergo dissociation and degradation. Subsequently, cobalamin is freed into the cytoplasm [74].

During the process in which holo-TC uptake receptors are broken down, the occurrence of CD320 on the cell surface is the direct result of its synthesis [75]. The level of the CD320 receptor is highly associated with the cell-division cycle and its presence on the cell surface is high in fast-growing cells, including cancer cells [74]. The impact of CD320 deficiency was tested in animal models with CD320 knock-out (KO) mice fed with a vitamin B_12_ deficient diet. Those animals showed increased MMA and homocysteine levels in the serum, as well as macrocytic anemic phenotype [76]. Neutrophil hypersegmentation is usually also a clinical hallmark of cobalamin uptake failure in patients [77]. Moreover, female mice with CD320 depletion and limited cobalamin intake exhibited infertility. Even though supplementation with cyanocobalamin resulted in a successful pregnancy, all litters died just after birth [76]. Other studies indicate that the knockdown of CD320 caused cognitive and learning disorders along with anxiety behavior corresponding to symptoms observed in humans, pointing out direct changes in the central nervous system [78]. It is suggested that observed alternation of DNA methylation in CD320 KO mice brains might cause neuron demyelination [79].

A single nucleotide polymorphism of CD320, rs2336573, leading to a glycine-to-arginine change has been identified in patients with lowered serum vitamin B_12_ levels [80]. Analogous outcomes occurred at a single codon deletion of c.262_264GAG (p.E88del). These observations have been linked with an increased MMA concentration in newborns [81]. However, in 2022, Pangilinan et al., using population data, estimated that approximately 85% of p.E88del homozygotes born in the same period did not have elevated propionylcarnitine (C3), suggesting that cobalamin metabolism in the majority of these infants with this genotype is unaffected. Thus, more studies are required to clarify the natural history of various *CD320* variants across patient populations [82].

#### 2.3.2. ATP Binding Cassette Subfamily D Member 4 (ABCD4) and the Lysosomal Cobalamin Transport Escort Protein (LMBD1)

Free vitamin B_12_ is released into the cytoplasm by an ATP (adenosine-5′- triphosphate) Binding Cassette Subfamily D Member protein (ABCD4, encoded by the gene of the same name), a 68.6 kDa exporter located on the lysosomal membrane [83]. ABCD4 consists of a transmembrane domain and a nucleotide-binding domain, which is involved in binding and hydrolysis of ATP [84]. It belongs to the family of ATP-binding cassette (ABC) transporters that mediate the transport of various substrates across the cell membrane against the concentration gradient. Most of the human ABC exporters are localized in a way that allows the substrate to access from the binding site, in a *cis*-acting manner. As revealed by cryo-electron microscopy (EM) studies, ABCD4 is a unique exporter, facilitating transfer in a *trans*-acting manner, thus allowing the transfer of vitamin B_12_ from the lysosome to the cytoplasm [85].

ABCD4 is a membrane endoplasmic reticulum (ER)-resident protein and requires translocation from the ER to the lysosome to facilitate vitamin B_12_ release. ABCD4 was found to colocalize with a 61.4 kDa membrane lysosomal cobalamin transport escort protein (LMBD1, encoded by gene *LMBRD1*), which localizes primarily to lysosomes [83,86]. Initially, LMBD1 was suggested to act as a lysosomal exporter of vitamin B_12_ [87]. However, later findings demonstrated that LMBD1 acts as an adaptor protein and mediates the translocation of ABCD4 from ER to the lysosomes [88]. Both proteins function in a coordinated manner and form a complex, as demonstrated by Surface Plasmon Resonance (SPR) [86].

Mutations in *ABCD4* and *LMBDR1* genes cause disease group cobalamine J and F diseases (cblJ, cblF), respectively [83,87]. At the cellular level, both errors are marked with a decreased function of methylmalonyl-CoA mutase and methionine synthase and an accumulation of free vitamin B_12_ in the lysosomes [89]. However, up to date, there is no clear biochemical or clinical distinction between cblJ and cblF. Only molecular genetic testing, either gene-targeted (RNA-sequencing and single-gene testing) or comprehensive (e.g., exome sequencing), was successfully applied to confirm each of the defects [90,91,92]. There is a wide spectrum of clinical presentations in both diseases, which may include hampered growth, developmental delay, developmental abnormalities progressive hyperpigmentation, cardiac defects, and hematologic impairments (such as macrocytic anemia, neutropenia thrombocytopenia, and pancytopenia). Neurological presentations may include general weakness, dizziness, delayed motor development, and mild intellectual disability [83,87,90,93].

#### 2.3.3. Methylmalonic Aciduria and the Homocystinuria Type C Protein (MMACHC)

Cobalamin that is released from lysosomes into the cytosol is available for two cytoplasmic proteins—methylmalonic aciduria and homocystinuria type C protein (MMACHC or CblC, encoded by *MMACHC* gene) and type d protein (MMADHC or CblD, encoded by *MMADHC* gene). The analysis of individuals with inborn errors of cobalamin metabolism revealed that the *MMACHC* gene is localized to the 1p34.1 region of chromosome 1 and consists of five exons. A study by Wang et al. suggested that c.609G > A, c.658_660delAAG, c.80A > G, and c.482 G > A mutations were the most common mutations in the Chinese population, while the most frequent mutations in the Caucasian population were c.271dupA, c.394C > T, and c.331C > T [94,95]. The *MMACHC* gene product consists of 282 amino acids and has a mass of 31.7 kDa [96]. *MMACHC* manifests a substantial degree of evolutionary conservation across mammalian species and exhibits high expression levels in the fetal liver, with its presence also identified in a broad spectrum of tissues, including the spleen, thymus, and bone marrow [96].

The *MMACHC* gene encodes a protein possessing both chaperone and enzyme functionalities. Its inactivation disrupts the reduction in incoming cobalamin molecules, which is necessary for the following synthesis of two essential cobalamin forms: methylcobalamin (MeCbl) and adenosylcobalamin (AdoCbl), which are cofactors for enzymes dependent on vitamin B_12_ [97]. The defects in MMACHC impact a stage occurring after the cellular absorption of cobalamin but preceding the synthesis of AdoCbl and MeCbl [96]. MMACHC catalyzes the reductive decyanation of cyanocobalamin (CNCbl or vitamin B_12_ in conjunction with a flavoprotein oxidoreductase) [98]. Interestingly, CblC also exhibits the remarkable capability to catalyze the nucleophilic displacement of the alkyl group by glutathione when presented with an alkylcobalamin. However, different forms of cobalamin are processed with a different efficacy by the intracellular system of enzymes and chaperons (e.g., better for GSCbl in comparison to other Cbl-forms [99]). This leads to considerable variation in the rates of the intracellular accumulation of those Cbls and, consequently, to different rates of the coenzyme appearance as established for HOCbl and CNCbl [100]. The intricate protein–protein interaction between MMACHC and MS is widely acknowledged for its pivotal role in regulating intracellular cobalamin metabolism. Notably, the interaction of MS inactive isoforms impedes MMACHC activity [101]. MMCHC is also essential in the conversion of MMA to succinyl-CoA [98].

Mutations in the *MMACHC* gene can result in a rare genetic disorder referred to as methylmalonic aciduria and homocystinuria type C (cblC). They demonstrate deficiencies in both vitamin B_12_-dependent enzymatic functions crucial in mammals—MS and MUT [98]. Thus, this disorder is characterized by the accumulation of both MMA and homocysteine (Hcy) in serum [98]. Individuals affected by the cblC disorder manifest severe neurological and systemic metabolic abnormalities [97], such as atypical hemolytic uremic syndromes (aHUS), decline in renal function, idiopathic neuropathies, spinal cord degenerations, ataxias, recurrent thrombosis, visual field defects, maculopathy, and optic disc atrophy [102]. Interestingly, recent studies suggest that the involvement of cobalamin and MMACHC might have a potential role in the oncogenic transformation of specific tumor subtypes. Melanomas and cell lines derived from melanoma often exhibit a higher occurrence of elevated methylation in the *MMACHC* promoter compared to other cancer types. This fact implicates the inactivation of *MMACHC* as the causative factor [97,103]. However, Bauer et al. highlighted that the defect in metabolism observed in MeWo-LC1 was unique and that decreased *MMACHC* expression was not a cause of methionine dependence in the other melanoma lines [104].

#### 2.3.4. Methylmalonic Aciduria and the Homocystinuria Type D Protein (MMADHC)

The *MMADHC* gene is associated with methylmalonic aciduria type D and homocystinuria and it is classified within the cblD complementation group [105]. The *MMADHC* gene encodes a protein consisting of 296 amino acids, with a molecular weight of 32.9 kDa. The MMADHC protein (also called CblD protein) exhibits a high degree of conservation across various mammalian species. The *MMADHC* gene encodes a robust N-terminal mitochondrial leader sequence along with a conserved vitamin B_12_-binding motif located at residues 81–86 [105]; however, a recent study has also shown cobalamin binding to a different region of the MMADHC protein [106]. It is present in both the mitochondria and cytoplasm, although the precise mechanisms that lead to this dual localization are not fully understood. The analysis of *MMACHC* and *MMADHC* gene expression patterns during mouse development unveils overlapping expression, particularly evident in the developing cardiac, respiratory, and nervous systems. It is suggested that these two proteins are co-expressed in all cells, as there is no discernible specificity for any particular organ, tissue, or cell type exclusively expressing *MMADHC*. Notably, *MMADHC* demonstrates expression in all cells of embryos, including those expressing *MMACHC* [107].

The physiological function of MMADHC is yet to be fully understood; however, it seems to play a role in the delivery of the cobalamin precursor to either cytoplasmic or mitochondrial sites of the production of the two active cobalamin forms, MeCbl and AdoCbl [108]. Plesa et. al., in 2011, employed a phage display to predict regions on MMADHC that bind to MMACHC. The study identified five distinct regions on MMADHC, with all aligning with residues C-terminal to Met116 [109] but not with the N-terminus (residues 1–61) [110]. MMADHC lacks any recognized enzymatic activity and it is suggested to be the first protein identified to repurpose the nitroreductase fold exclusively for protein–protein interactions [111].

The cblD complementation group can be linked to isolated methylmalonic aciduria (cblD-MMA), isolated homocystinuria (cblD-HC), or a combination of both methylmalonic aciduria and homocystinuria (cblD-MMA/HC) [112]. Individuals characterized by cblD-MMA have at least one altered allele that induces a premature stop codon in the N-terminal region of the protein. In contrast, those with cblD-HC possess at least one missense mutation, resulting in amino acid substitutions located in the C-terminal part of the protein [108]. The clinical presentation indicates that patients with cblD-MMA/HC and cblD-HC exhibit neurological and hematological symptoms. In contrast, cblD-MMA patients experience respiratory distress, hyperammonemia, and neurological symptoms [105,112,113].

### 2.4. Vitamin B_12_-Dependent Intracellular Enzymes

#### 2.4.1. Methionine Synthase (MS)

Methionine synthase (MS, encoded by gene *MTR*) is a big ~140 kDa monomeric protein found in the cytosol [114] and it consists of four domains. In bacteria, the N-terminal site, dependent on the zinc ions, binds and activates Hcy while the following domain has a high affinity to methyltetrahydrofolate and might affect its activity [115,116]. The third module is vitamin B_12_-dependent and crucial in methyl group transfer from 5-methyltetrahydrofolate to Hcy with the production of tetrahydrofolate and methionine [116]. Finally, the C-terminal domain binds S-adenosylmethionine for reductive activation of MS [117], when cobalamin cofactor undergoes oxidation process [117]. The human enzyme displays a 58% identity with MS from *Escherichia coli* (*E. coli*) [118], which is one of the biggest proteins with 1227 residues and a molecular weight of ~136 kDa [119]. The remarkable conservation of amino acid residues across proteins indicates a high degree of similarity in properties and structures between enzymes from both humans and *E. coli*. Consequently, the information gleaned from the *E. coli* enzyme is presumed to apply to its human counterpart [118]. However, a crucial divergence is the lack of flavodoxin in mammals, a pivotal component in the reductive reactivation process observed in bacterial models [120].

In mice models, full depletion of the *MTR* gene caused pregnancy failure [114]. In proliferating cells of the skin and intestinal epitheliums, as well as in rat intestine crypts and proliferating Caco-2 cells (human epithelial colorectal adenocarcinoma cell line), there is a consistent increase in the transcription, protein expression, and activity of MS. Additionally, MS activity correlates with DNA methylation in rat intestine villi [121]. Moreover, in situ hybridization indicated that the human *MTR* gene is found on the 1q43 chromosome region [118] and its missense mutation or deletion of three base pairs was identified in the cblG group of patients with cobalamin metabolism disorder [122]. However, the second form of this disorder has also been recognized as “methylcobalamin deficiency cblE type” (cblE) and it is believed to arise from mutations occurring at distinct genetic loci [123]. This rare autosomal disease is associated with homocysteinemia, homocystinuria, and hypomethioninemia [122] and patients usually exhibit symptoms within the initial two years of their lives [124]. Reduced MS activity also leads to an elevation in the catalytic protein phosphatase 2A (PP2A), resulting in an imbalance in the phosphorylation/methylation of nucleocytoplasmic RNA binding proteins. This leads to compromised energy metabolism and neuroplasticity linked to inborn errors of Cbl metabolism (IECM) [125].

#### 2.4.2. Methylmalonyl-CoA Mutase (MCM)

Methylmalonyl coenzyme A mutase (MCM, encoded by gene *MMUT*) is an enzymatic protein found in mitochondrial matrix with a ~78 kDa molecular weight [126]. The structure of MCM is highly conserved evolutionally; thus, this AdoCbl-dependent enzyme may be found in both eukaryotes and prokaryotes [126] but not plants [127]. MCM takes part in converting carbon skeletons of odd-chain fatty acids, cholesterol, and amino acids such as threonine, methionine, valine, and isoleucine into succinyl-CoA for further utilization in the tricarboxylic acid (TCA) cycle [128].

It has to be mentioned that the proper MCM activity depends on AdoCbl, the synthesis of which involves chaperones encoded by the *cblA* and *cblB* genes. The *cblA* gene encodes a GTPase methylmalonic aciduria type A (MMAA) and *cblB* encodes adenosyltransferase (ATR), with each playing unique roles in cofactor assimilation, loading, and repair of MCM [126]. ATR, also known as MMAB for its role in methylmalonic aciduria type B, is a multifunctional protein acting as both an enzyme and a chaperone in mitochondrial transport [129]. Its adenosyltransferase activity converts inactive cobalamin into AdoCbl and inorganic triphosphate in the presence of a reductant and ATP. Some studies show that inactivation of MCM occurs when AdoCbl is oxidized to HOCbl [130]. The inactivation of MCM occurs when the active adenosyl radical generated during the enzyme catalytic cycle reacts with a molecule other than the substrate, inactivating the enzyme. However, the formation of the MMUT-methylmalonic aciduria type A (MMAA) protein complex with MCM reduces the rate of oxidized cofactor formation, protecting enzyme activity. In detail, the enzyme is inactivated until the damaged cobalamin is removed and replaced by MMAA [131].

Human MCM is encoded by the *MMUT* gene, situated as a lone copy on chromosome 6 (6p21.3), and composed of 13 exons [132]. Its mutation leads to methylmalonic acidemia—a metabolic autosomal disease. Patients with acidemia suffer from vomiting followed by dehydration, muscle weakness, lethargy, mental incapacity, and, rarely, to death when not treated [133].

Two subgroups of MCM mutation have been recognized so far: the mut^−^ defect, characterized by residual activity in the presence of high concentrations of AdoCbl, and the mut^0^ defect, with complete loss of MCM activity [134]. Distinguishing between mut^−^ and mut^0^ subgroups can be challenging at times. Certain mut^−^ mutations may only impact the apparent Michaelis constant (K_m_), while others can influence both the apparent K_m_ and the maximum velocity of an enzymatic reaction (V_max_) [133].

## 3. Vitamin B_12_ Deficiency

### 3.1. Epidemiology and Symptoms of Vitamin B_12_ Deficiency

The prevalence of vitamin B_12_ deficiency in the United States (US) ranges from 2% to 15%, depending on the biomarker (e.g., total/active vitamin B_12_ metabolites or combined index of vitamin B_12_ status) and its cut-off used, as well as on the age, gender, and ethnicity [135,136]. An analysis of National Health and Nutrition Examination Survey data from 2007 to 2018 revealed that about 3.6% of all adults aged 19 and older have a vitamin B_12_ deficiency (in the US, it is defined as serum vitamin B_12_ levels below 200 pg/mL (148 pmol/L)). The deficiency rate increases to 3.7% in individuals aged 60 and older. However, vitamin B_12_ insufficiency (defined as serum levels below 300 pg/mL (221 pmol/L)) is more prevalent, affecting approximately 12.5% of all adults aged 19 and older and 12.3% of those aged 60 and older [137]. During pregnancy, serum vitamin B_12_ levels often decline, sometimes dropping to subnormal levels, but typically return to normal after delivery [138]. Furthermore, vitamin B_12_ deficiency due to pernicious anemia is more common in people of northern European ancestry than people of African descent [139,140].

Food-bound cobalamin malabsorption (FBCM) is among the frequent causes of vitamin B_12_ deficiency [141]. FBCM is characterized by a hindered liberation and absorption of cobalamin from food sources [142]. Impaired release of cobalamin from its gastrointestinal transport proteins is commonly seen in conditions like gastritis or during medication use, reducing hydrochloric acid production. Milder symptoms may persist due to impaired IF-dependent transport functionality [143]. Proton pump inhibitors and histamine H2-receptor antagonists heighten the risk of future cobalamin deficiency, while cessation of medication reduces the risk. For example, the widespread use of acid-suppressing medications may lead to undiagnosed cobalamin deficiency [144].

The effects of vitamin B_12_ deficiency are mainly seen in the blood and nervous system. Thus, the classic manifestation was first identified as pernicious anemia [145]. Since then, the spectrum has shifted considerably, starting with the recognition that neurological manifestations (ataxia, cognitive decline leading to dementia, or psychiatric disorder) often predominate and can occur in the absence of hematological disorders [146]. Anemia is caused by disruption of DNA synthesis in a hematopoietic system, predominantly erythroid precursors. It is caused by a “folate trap”, i.e., when the low activity of MS (caused by a low MeCbl coenzyme level) is not sufficient to liberate free tetrahydrofolate from its inactive methyl-complex. Neurological complications are associated with impaired methylation of myelin lipids caused by insufficient production of S-adenosyl methionine (mediated by MeCbl). Such insufficiency damages the isolating myelin sheath of axons [31,147,148]. Moreover, lack of MeCbl or MTR inhibition leads to hyperhomocysteinemia linked with inflammation, oxidative stress, and microvascular disease that may exacerbate neurological symptoms [149].

In addition to the symptomatic presentation of vitamin B_12_ deficiency, subclinical cobalamin deficiency (SCCD) presents a condition characterized by biochemical aberrations in the absence of clinical signs [141,150]. SCCD is encountered more commonly than the overt deficiency and it often originates from FBCM [151,152]. Currently, SCCD is perceived as a transient state without overt cobalamin depletion, necessitating exploration into remediable etiologies. Patients exhibiting serum cobalamin levels between 110 and 148 pmol/L should be reexamined by other markers to the test (e.g., MMA) and undergo retesting within one to two months; those exhibiting normalization need not undergo further assessment [153]. Persistent low levels may warrant low-dose oral cobalamin supplementation alongside anti-IF antibody analysis. Neurological manifestations should prompt reassessment by a healthcare provider. Positive anti-IF antibody titers necessitate treatment for pernicious anemia, while negative results mandate reevaluation after three to four months [154]. Additional biochemical assessments are warranted if cobalamin levels remain consistently low.

### 3.2. Diagnosis and Treatment of Vitamin B_12_ Deficiency

The assessment of vitamin B_12_ status typically relies on measurements of its serum or plasma levels. Laboratories often define subnormal values as below 200 or 250 pg/mL (148 or 185 pmol/L). Typically, a value significantly below the lower limit of the reference range suggests probable vitamin B_12_ deficiency, while a value well above probably indicates its adequate status. However, exceptions arise in patients with pernicious anemia who have circulating antibodies against IF; their vitamin B_12_ levels may appear falsely normal despite deficiency, supposedly because of the immunological interference from anti-IF antibodies with the test setup [155]. Active vitamin B_12_ bound to transcobalamin (holotranscobalamin) theoretically offers increased sensitivity for detecting vitamin B_12_ deficiency compared to total serum vitamin B_12_ levels. Although the measurement of holotranscobalamin is gradually becoming available in clinical settings, it has demonstrated only marginal improvement over total vitamin B_12_ levels as a biomarker for deficiency [156,157].

Another valuable and highly sensitive marker of vitamin B_12_ status is the serum MMA level, with a threshold greater than 0.271 µmol/L indicative of deficiency [11]. However, MMA levels can be elevated due to renal insufficiency and tend to be higher in older adults. Genetic influences on MMA cutoffs have also been highlighted by genome-wide association studies. A common polymorphism (rs291466) in *HIBCH*, involved in valine catabolism, is prevalent (minor allele frequency 0.43) and homozygotes for the allele show 46% higher plasma MMA levels that potentially impact its efficacy of this metabolite as a vitamin B_12_ status indicator [158]. Besides MMA concentration, total plasma Hcy levels also rise rapidly to levels above 15 µmol/L, with declining vitamin B_12_ status. However, Hcy levels lack specificity, being influenced by factors such as folate levels or renal function decline [159]. To overcome these challenges, different combinations of the aforementioned markers are frequently used in analysis [160] and references.

Other biochemical parameters that might be useful in diagnosing vitamin B_12_-related disorders include methionine. Indeed, in remethylation disorders, methionine levels might be suppressed but, more frequently, they remain stable. Thus, measuring methionine may help differentiate remethylation disorders from classical homocystinuria in cases with elevated Hcy [161]. Remethylation defects are linked with impaired remethylation of homocysteine to methionine and thus lead to the accumulation of homocysteine and perturbation of numerous methylation reactions [162]. All genetic remethylation defects result in reduced methionine synthase activity due to various factors: decreased function of the enzyme itself or its associated enzyme, methionine synthase reductase; insufficient production of the cofactor methylcobalamin; or disrupted availability of the substrate methyl-tetrahydrofolate (MTHF) [163]

Optimal management of vitamin B_12_ deficiency requires identifying its cause, particularly differentiating between its low intake and malabsorption. The Schilling test, once the gold standard, is now rarely used due to the lack of radiolabeled vitamin B_12_ availability and cost constraints. The CobaSorb test, measuring serum holotranscobalamin levels of post-vitamin B_12_ dosing, is another opportunity [164,165,166]. Other tracers (e.g., low radioactive ^14^C- and nonradioactive ^13^C-labeled vitamin B_12_) are currently discussed as feasible alternatives to ^57^Co-labeled vitamin B_12_ of the Schilling text [167,168]. Alternative diagnostic approaches include detecting autoantibodies for pernicious anemia and assessing for atrophic gastritis during upper endoscopy [169]. While a negative result for anti-IF autoantibodies does not exclude pernicious anemia, a positive result is highly specific. However, a positive result for anti-H^+^/K^+^ ATPase autoantibodies lacks specificity, as it may occur in patients without pernicious anemia [170,171]. In pediatric cases, consideration of inborn errors of cobalamin metabolism via complementation phenotyping or genetic analysis is crucial for diagnosis. These tests help differentiate between different genetic causes, aiding in tailored treatment approaches for children with vitamin B_12_ deficiency [12,172].

Treatment for vitamin B_12_ deficiency varies depending on the underlying cause. In 2021, The British Society for Haematology (BSH) updated guidance on vitamin B_12_ replacement [173]. For non-diet-related deficiency, such as in pernicious anemia or other conditions, hydroxocobalamin 1 mg intramuscular every 2–3 months for life is recommended, though the optimal time intervals might significantly vary from patient to patient. For diet-related deficiency, individuals can either orally intake cyanocobalamin (50–150 micrograms daily) between meals or receive a twice-yearly hydroxocobalamin 1 mg injection. In cases of dietary deficiency, treatment duration may vary, with vegans potentially requiring lifelong treatment.

### 3.3. Long-Term Consequences of Vitamin B_12_ Deficiency

Vitamin B_12_ deficiency, owing to its pivotal role in cellular metabolism, can mimic a diverse array of clinical presentations as listed above. However, a manifestation of these clinical outcomes is relatively late. It results in the development of SCCD, which may have severe consequences, including increased risk of neurodegenerative and cardiovascular diseases or, possibly, enhanced cancer progression due to the accumulation of MMA according to [174].

In vegetarians, deficiency of vitamin B_12_ was mostly found to be associated with depression and adverse neurological function. Berkins et al. highlighted that the dietary intake of vitamin B_12_ might affect brain structure [175]. Interestingly, supplementation of vitamin B_12_ along with anti-depressant therapy greatly improved depressive symptoms [176]. Other studies conclude that cobalamin deficiency may have some similarities with multiple sclerosis. In both conditions, destroyed myelin sheaths release glutamate, which is known to possess exotoxicity [177]. It is well known that cognitive decline often coincides with a high prevalence of vitamin B_12_ deficiency, although causality in these correlations is not fully established [178]. Furthermore, while the actual percentage of reversible dementia in individuals with vitamin B_12_ insufficiency is deemed low, impaired vitamin B_12_ metabolism could potentially serve as one of several factors influencing the onset and progression of cognitive decline and dementia [179]. It could be caused by reduced methylation capacity, elevated Hcy levels, and the role of B vitamins in upholding the integrity of the blood–brain barrier [180]. Some researchers propose that it may act as a predisposing factor for Alzheimer’s disease [181]. Additionally, it is associated with various other neurological conditions including Wernicke’s encephalopathy, subacute combined degeneration of the spinal cord, and peripheral neuropathy [182].

It is well known that hyperhomocysteinemia, strongly linked to vitamin B_12_ deficiency, is a prominent risk factor for atherosclerotic vascular diseases affecting coronary, cerebral, and peripheral vessels, as well as arterial and venous thromboembolism [183]. The adverse effects of Hcy on the vascular endothelium and coagulation cascade, along with its procoagulant properties, encompass reduced binding of antithrombin III to endothelial heparan sulfate, heightened affinity between lipoprotein (a) and fibrin, increased tissue factor activity in endothelial cells, and inhibition of factor V inactivation by activated protein [184]. Supplementation with vitamin B_12_ led to the resolution of both clinical and biological abnormalities in all participants [185]. However, whether vitamin B_12_ deficiency directly influences thrombosis or acts through hyperhomocysteinemia or other factors remains to be fully understood [186]. Lifestyle factors like smoking, BMI, and physical activity may also contribute to the association between hyperhomocysteinemia and thromboembolism [187].

Interestingly, other compounds that are strongly linked to vitamin B_12_ deficiency (e.g., MMA) have been recently identified as oncometabolites. MMA is a metabolite of methylmalonyl coenzyme A; its level is increased because of impaired conversion to succinyl coenzyme A mediated by a coenzyme form of vitamin B1_2_ (AdoCbl) [188]. A study published in 2020 by Gomes et al. revealed that MMA levels increase in the serum of older individuals and may have a function as a mediator of tumor progression. Indeed, treatment of adenocarcinoma human alveolar basal epithelial (A549) cells with MMA induced a complete pro-aggressive epithelial to mesenchymal transition-like phenotype with a decline in E-cadherin and a concurrent increase in fibronectin and vimentin [174]. Additionally, two years later, Gomes et al. demonstrated that the accumulation of MMA accelerates cancer cell aggressiveness in breast and lung cancers [174,189]. Intriguingly, in the context of breast cancer, the blockade of other metabolic pathways involving MMA or the activation of MMA production, coupled with vitamin B_12_ intracellular deficiency, resulted in enhanced tumor metastasis [174,189]. Recently, MMA was also found to promote metastasis in colon tumor models [190]. However, the direct effect of vitamin B_12_ deficiency on cancer onset, prognosis, and progression remains unresolved due to many inconsistent studies [191,192,193,194,195,196].

## 4. Discussion

In this review, we consolidate existing knowledge regarding the proteins involved in vitamin B_12_ metabolism and explore their role in the cellular processes as well as the impact of their dysfunction on overall metabolic integrity. Several proteins listed in Table 1, including transcobalamin, haptocorrin, Intrinsic Factor, cubilin, and amnionless, are already utilized in the diagnosis of vitamin B_12_ deficiency. However, other proteins are primarily investigated at the gene level due to their intracellular localization or low detection levels in serum, limiting their use in routine diagnostics. Nonetheless, the levels of some of these proteins may harbor novel information concerning the risk of complications associated with vitamin B_12_ deficiency.

In 2018, Tseng et al. established that mice models demonstrated that a loss of single allele *Lmbd1* would result in ventricular hypertrophy, which was accompanied by enhanced myocardial glucose uptake [197]. Moreover, a microarray screening study revealed a 25% reduction in LMBD1 mRNA levels in the septa of 13 heart transplant patients with dilated cardiomyopathy compared to non-failing hearts [198]. This clinical observation, coupled with the findings from mice studies, suggests that diminished expression of LMBD1 protein may play a role in the development of cardiomyopathy. Thus, the single nucleotide polymorphism sites associated with decreased expression of the human *LMBRD1* gene evaluation may not only indicate the cause of vitamin B_12_ deficiency but also prognose a risk of onset of cardiovascular diseases.

Interestingly, the diminished expression of other proteins involved in vitamin B_12_- has been noted in the brains of multiple sclerosis patients, highlighting the role of this protein in proper central nervous system function [199]. It was accompanied by worsening the disease by both CD320 genetic deletion and vitamin B_12_ deficiency [199]. These findings highlight a plausible connection between vitamin B_12_ deficiency and the progression of pathology in patients with multiple sclerosis. One may conclude that vitamin B_12_ deficiency caused by CD320 disruption might be linked with an increased risk of neurodegenerative diseases; thus, its evaluation might be an interesting prognostic parameter.

Another protein linked with vitamin B_12_ metabolism that exhibits multifunctional properties is MRP1. MRP1 transports a wide range of therapeutic agents as well as various physiological substrates and may contribute to drug resistance development in several cancers, including lung, breast, and prostate cancers, as well as childhood neuroblastoma [200]. MRP1’s role as a glutathione transporter creates a unique vulnerability in cancer cells with high MRP1 expression, providing a potential therapeutic opportunity for targeting cancer. Direct pharmacological inhibition of MRP1 shows promise and is extensively tested in preclinical and clinical studies [201,202,203]. However, non-selective MRP1 inhibition could potentially disrupt vitamin B_12_ metabolism and lead to its deficiency. Therefore, there is an emerging need to investigate the effects of MRP-1 inhibition on vitamin B_12_ metabolism during therapy.

Interestingly, it has been noted that the utilization of the common drug metformin in diabetic patients is linked to potential malabsorption and deficiency of vitamin B_12_ [204,205]. Numerous observational studies and meta-analyses have underscored a significant correlation between metformin usage and vitamin B_12_ deficiency [206]. Patients undergoing metformin therapy display reduced absorption of B_12_, leading to decreased levels of serum total vitamin B_12_ and holoTC (“active vitamin B_12_”). This phenomenon is attributed to calcium-dependent antagonism in the ileal membrane, which may be mitigated through calcium supplementation [207]. Given these findings, it is strongly advised that individuals using metformin undergo regular monitoring of their vitamin B_12_ levels. This precaution is crucial as prolonged metformin usage may deplete hepatic vitamin B_12_ reserves [208].

## 5. Conclusions

Despite the initiation of studies on vitamin B_12_ as early as in the 1948s, our understanding of its biochemical alterations remains incomplete. Over the past decade, there has been a notable surge in research attention directed toward vitamin B_12_ biochemical, physiological, and clinical facets. Surprisingly, some recent studies suggested that vitamin B_12_ may affect gene expression, cellular plasticity, and tissue repair [178,209]. However, the specific molecules and cellular changes mediated by these alterations are still not fully elucidated.

Besides the fact that vitamin B_12_ deficiency leads to the presence of serious clinical manifestations, numerous studies have highlighted the correlation between low vitamin B_12_ levels and the increased risk of civilization diseases. Importantly, some of the proteins involved in vitamin B_12_ metabolism may act as moonlighting proteins and their pharmacological modification already used in the treatment of other clinical stages may implicate also proper vitamin B_12_ metabolism.

Hence, there is a pressing need for more basic science studies to unravel the vitamin B_12_ metabolism complexity that may lower the risk of vitamin B_12_ deficiency in clinical studies as well as potentially identify novel diagnostic parameters that may contribute to evaluating the risk of vitamin B_12_ deficiency consequences.

## Figures and Tables

**Table 1:** Proteins are involved in vitamin B_12_ metabolism.

Protein		Shortname	Gene	Type	Function	Pathology	Ref.
Haptocorrin, Transcobalamin I		HC, TC-I	*TCN1*	Glycoprotein	Binding vitamin B_12_ in saliva and blood, transferring the vitamin to the stomach; Protection of vitamin B_12_ from hydrolysis in the acidic pH of the stomach.	The inability of HC degradation and release of the vitamin from the vitamin B_12_–HC complex (caused by deficiency of pancreatic proteases) may lead to vitamin B_12_ malabsorption.	[15,16,17,18,19,20]
Intrinsic factor		IF	*GIF* or *CBLIF*	Glycoprotein	Binding vitamin B_12_ in the duodenum.	The lack of IF in the condition of *H. pylori* infection or AIG leads to long-lasting vitamin B_12_ deficiency due to the inability to deliver vitamin B_12_ to the cells within the ileum.	[15,16,21]
Cubam receptor	Cubilin	CUBN	*CUBN*	Multiligand apical membrane receptor	Interaction with IF; Docking to transmembrane protein AMN; Forming with AMN a receptor specific for IF–vitamin B_12_ internalization.	Damage of this cubam results in malabsorption of vitamin B_12_ and the development of Imerslund–Gräsbeck syndrome (IGS), a rare autosomal recessive disorder that appears in childhood, characterized by megaloblastic anemia.	[32,33,36,37]
	Type-1 transmembrane protein amnionless	AMN	*AMN*		Clathrin-mediated internalization; Anchoring Cubilin; Forming with CUBN a receptor specific for IF–vitamin B_12_ internalization.		
Multidrug resistant protein 1		MRP1, ABCC1	*ABCC1*	ATP-binding cassette (ABC) transporters family	Effluxing vitamin B_12_ from the intestinal epithelium to the blood circulation.	Mutations in the gene *ABCC1* were detected in some patients with vitamin B_12_ malabsorption; however, it is an unlikely cause of Cbl deficiency in humans.	[46,47,52,54]
Transcobalamin		TC, TC-II	*TCN2*	β-globulin protein	Binding vitamin B_12_ in the bloodstream; Facilitated distribution of vitamin B_12_ into all cells in the body.	Mutations in the gene can lead to intracellular cobalamin depletion, causing a rare multisystemic disorder characterized by autosomal recessive inheritance.	[55,59,60,65,66,67]
Receptor for cluster of differentiation 320, Receptor for cobalamin-saturated transcobalamin		CD320, TCblR	*CD320*	Low-density Lipoprotein receptor protein family	Endocytosis of the TC–cobalamin complex.	Inconsistent studies: in 2010, Quadros et al. associated elevated MMA in five newborns with a mutation in *CD320*, while in 2022 Pangilinan et al. estimated that approximately 85% of infants with this mutation have unaffected cobalamin metabolism.	[68,69,70,72,73,80,81,82]
ATP-binding cassette sub-family D member 4		ABCD4, CblJ protein	*ABCD4*	Exporter protein	Transport of free vitamin B_12_ into the cytoplasm.	Mutations are related to disease groups cblJ and cblF. At the cellular level, both errors are marked with a decreased function of MMUT and MTR and an accumulation of free vitamin B_12_ in the lysosomes.	[83,86,87,88,90,93]
Lysosomal cobalamin transport escort protein		LMBD1, CblF protein	*LMBRD1*	Adaptor protein, chaperone	Mediates the translocation of ABCD4 from ER to the lysosomes.		
Methylmalonic aciduria and homocystinuria type C protein		MMACH, CblC protein	*MMACHC*	Chaperone, enzyme	Potentially receiving the Cbl cargo upon its release from the lysosomal compartment; Catalyzing the reductive decyanation and delkylation of cobalamin.	Mutations result in a rare genetic disorder referred to as methylmalonic aciduria and homocystinuria type C (cblC); deficiencies in both vitamin B_12_-dependent enzymatic functions MTR and MMUT—accumulation of both MMA and Hcy in serum.	[94,96,97,98,102]
Methylmalonic aciduria and homocystinuria type D protein		MMADHC, CblD protein	*MMADHC*	Probably enzyme	Delivers Cbl-precursor to the production sites of MeCbl and AdoCb.	The cblD complementation group can be linked to isolated methylmalonic aciduria (cblD-MMA), isolated homocystinuria (cblD-HC), or a combination of both methylmalonic aciduria and homocystinuria (cblD-MMA/HC).	[105,107,108,111,112,113]
Methionine synthase		MTR, MS	*MTR*	Cytoplasmic enzyme	Vitamin B_12_-dependent transfer of methyl group from 5-methyltetrahydrofolate to homocysteine with the production of tetrahydrofolate and methionine.	Missense mutation or deletion of three base pairs was identified in the cblG group of patients with cobalamin metabolism disorder and methylcobalamin deficiency cblE type, rare autosomal diseases associated with homocysteinemia, homocystinuria, and hypomethioninemia.	[81,82,83,84,86,87,115,123,124]
Methylmalonyl-CoA mutase		MMUT, MCM, MUT	*MMUT*	Mitochondrial enzyme	Vitamin B_12_-dependent conversion methylmalonyl-CoA to succinyl-CoA.	Mutation leads to methylmalonic acidemia.	[88,89,90,92,128,131]
